# Supplementary material for: Validating anthropogenic threat maps as a tool for assessing river ecological integrity in Andean–Amazon basins
Source: PeerJ. 2019 Nov 20;7:e8060. doi: 10.7717/peerj.8060 (PMC6874857; doi:10.7717/peerj.8060)
Supplement: Supplemental Information 1 — When normalized on a scale of 0–1, a score of 1 represents values linked to higher ecological integrity. [file peerj-07-8060-s001.docx]

| Ecological Integrity Component | Parameters |
| --- | --- |
| (i) Biotic community composition. | Based on Average Score Per Taxa (∑ tolerance index of families/ number of families found at site). Scores were normalized on a scale of 0-1. |
| (ii) Habitat quality: average of normalized scores for Riparian Integrity and Fluvial Habitat Quality. | *Riparian Integrity*. Based on the QBR-Am Index, which grades the quality of the riparian habitat on a scale of 0-100, a score of 100 represented higher riparian habitat quality. QBR-Am scores were normalized on a scale of 0-1. |
|  | *Fluvial Habitat Quality*. Based on the IHF-Am Index, which grades the condition of the fluvial habitat from 0-100, a score of 100 represented higher fluvial habitat quality. IHF-Am scores were normalized on a scale of 0-1. |
| (iii) Physical-chemical characteristics: average of normalized scores for conductivity, pH, temperature and dissolved oxygen. | *pH.* The absolute difference between observed values and predicted values, which was normalized on a scale of 0-1. pH values that presented the highest variation from the references values received a lower score. |
|  | *Conductivity (μS/cm).* The absolute difference between observed values and predicted values, which was normalized on a scale of 0-1. Conductivity values that presented the highest variation from the references values received a lower score. |
|  | *Temperature (ºC).* The absolute difference between observed values and predicted values, which was normalized on a scale of 0-1. Observed values that were within a ±2°C range from the references values were considered to represent higher water quality. Temperature values outside that range were scored depending on their proximity to the reference value. |
|  | *Dissolved Oxygen (mg/L)*. The difference between measured data and expected data, which was normalized on a scale of 0-1. Observed values that were above the references levels, which for the sites at lower altitudes (< 600 m) are above 2 mg/L and for sites at higher altitudes are above 6 mg/L, were considered to represent high water quality. |
| **Ecological Integrity Index** | Average of the normalized scores of the 3 components. |
