# Supplementary material for: Validating anthropogenic threat maps as a tool for assessing river ecological integrity in Andean–Amazon basins
Source: PeerJ. 2019 Nov 20;7:e8060. doi: 10.7717/peerj.8060 (PMC6874857; doi:10.7717/peerj.8060)
Supplement: Supplemental Information 2 [file peerj-07-8060-s002.docx]

#### Script developed for the study "Validating anthropogenic threat maps as a tool for assessing

# river ecological integrity in Andean-Amazon basins". This script contains basic functions

# used or developing and validating empirical models that link the ecological integrity of rivers

# to threat and envirionmental maps

### 1. Load packages ###

if (!require("pacman")) install.packages("pacman")

pacman::p_load(readxl, MuMIn, caret, texreg, stargazer, dplyr, boot)

### 2. Data preparation ###

## 2a. Data reading

setwd("directory") # indicate directory

data <- read_excel("Appendix C.xlsx",1)

## 2b. Selection of the columns with the response and predictor variables

data <- data[,c(7:20)]

### 3. Global model ###

GlobalModel <- glm(`Ecological Integrity Index` ~., data, family = "gaussian")

summary(GlobalModel)

### 4. Correlation matrix for model selection ###

## 4a. Correlation function

is.correlated <- function(i, j, data, conf.level = .95, cutoff = .70, ...) {

if(j >= i) return(NA)

ct <- cor.test(data[, i], data[, j], method = "spearman", conf.level = conf.level, ...)

ct$p.value > (1 - conf.level) || abs(ct$estimate) <= cutoff

}

vCorrelated <- Vectorize(is.correlated, c("i", "j"))

## 4b. Applying the correlation function to our data (excluding the response variable)

data_m = data

data_m = data_m[,-1]

data_m =as.data.frame(data_m)

smat <- outer(1:ncol(data_m), 1:ncol(data_m), vCorrelated, data = data_m)

nm <- colnames(data_m[1:ncol(data_m)])

dimnames(matrix) <- list(nm, nm)

matrix

### 5. Selection of the best models according to AICc Akaike Information Criterion ###

## 5a. Evaluation of all possible sub-model (excluding those with correlated variables), based

# on different combination of predictor variables

options(na.action = "na.fail")

Selection <- dredge(GlobalModel, rank = "AICc", m.lim=c(1,7), subset = matrix)

## 5b. Exploring the three best models, according to the lowest AICc

screenreg(extract(subset(Selection, 1:3, recalc.weights = TRUE)), digits = 5)

## 5c.Extraction of the best model

bestmodel<-get.models(Selection, 1)[[1]]

summary(bestmodel)

# 5d. Evaluation of the best model

glm.diag.plots(bestmodel)

### 6. k fold crossvalidation for the best model

## 6a. Settings for the cross validation: seven groups, 20 repetitions

ctrl <- trainControl(method = "repeatedcv", number=7, repeats=20)

set.seed(2017)

## 6b. Cross validation to the best model parameters, using as example our best model for the scenario (b).

# Note: Replace these predictors with those of the model under evaluation

NewModel <- train(`Ecological Integrity Index` ~

`Human settlements` +

`Oil activities` +

Elevation_scaled +

Roads +

Slope,

method = "glm", trControl = ctrl, data = data)

## 6c. New coefficientes for the best model after the cross validation

NewModel$finalModel$coefficients

## 6d. Predictive power of the best model after cross validation

NewModel$results$Rsquared # Mean Rsquared

NewModel$results$RsquaredSD #Standard deviation
