# Supplementary material for: Validating anthropogenic threat maps as a tool for assessing river ecological integrity in Andean–Amazon basins
Source: PeerJ. 2019 Nov 20;7:e8060. doi: 10.7717/peerj.8060 (PMC6874857; doi:10.7717/peerj.8060)
Supplement: Supplemental Information 4 [file peerj-07-8060-s004.docx]

**Appendix D.** Extended Results

**Table 1**. Ranking of models for modeling scenario (a): prediction of ecological integrity at a local scale, using human threat variables as predictors.

|  | **Model 1** | **Model 2** | **Model 3** | **Model 4** | **Model 5** | **Model 6** | **Model 7** | **Model 8** |
| --- | --- | --- | --- | --- | --- | --- | --- | --- |
| (Intercept) | 0.738^***^ | 0.736^***^ | 0.740^***^ | 0.728^***^ | 0.741^***^ | 0.740^***^ | 0.738^***^ | 0.737^***^ |
|  | (0.010) | (0.010) | (0.011) | (0.009) | (0.011) | (0.011) | (0.010) | (0.010) |
| Oil activities | -0.106 |  | -0.113 | -0.148^*^ | -0.099 |  | -0.100 |  |
|  | (0.066) |  | (0.066) | (0.063) | (0.067) |  | (0.067) |  |
| Human settlements | -0.145^*^ | -0.176^**^ | -0.135^*^ | -0.161^*^ | -0.143^*^ | -0.171^**^ | -0.142^*^ | -0.169^**^ |
|  | (0.066) | (0.064) | (0.067) | (0.066) | (0.066) | (0.064) | (0.066) | (0.064) |
| Roads | -0.080 | -0.102^*^ | -0.080 |  | -0.084^*^ | -0.106^**^ | -0.078 | -0.098^*^ |
|  | (0.042) | (0.039) | (0.042) |  | (0.042) | (0.040) | (0.042) | (0.040) |
| Aquiculture |  |  | -0.044 |  |  |  |  |  |
|  |  |  | (0.046) |  |  |  |  |  |
| Water withdrawals |  |  |  |  | -0.235 | -0.303 |  |  |
|  |  |  |  |  | (0.322) | (0.320) |  |  |
| Thermoelectric |  |  |  |  |  |  | -0.033 | -0.048 |
|  |  |  |  |  |  |  | (0.059) | (0.058) |
| Log Likelihood | 136.070 | 134.747 | 136.550 | 134.207 | 136.344 | 135.206 | 136.236 | 135.091 |
| AICc | -261.692 | -261.197 | -260.468 | -260.118 | -260.057 | -259.965 | -259.841 | -259.735 |
| Delta | 0.000 | 0.495 | 1.224 | 1.574 | 1.635 | 1.727 | 1.851 | 1.958 |
| Weight | 0.227 | 0.177 | 0.123 | 0.103 | 0.100 | 0.096 | 0.090 | 0.085 |
| Num. obs. | 140 | 140 | 140 | 140 | 140 | 140 | 140 | 140 |
| ^***^p < 0.001, ^**^p < 0.01, ^*^p < 0.05 | | | | | | | | |

**Table 2**. Ranking of models for modeling scenario (b): prediction of ecological integrity at a local scale, using human threat and environmental variables as predictors.

|  | **Model 1** | **Model 2** | **Model 3** |
| --- | --- | --- | --- |
| (Intercept) | 0.762^***^ | 0.775^***^ | 0.763^***^ |
|  | (0.019) | (0.027) | (0.019) |
| Elevation | -0.176^***^ | -0.182^***^ | -0.174^***^ |
|  | (0.045) | (0.046) | (0.045) |
| Oil activities | -0.136^*^ | -0.127 | -0.139^*^ |
|  | (0.063) | (0.064) | (0.063) |
| Human settlements | -0.165^*^ | -0.147^*^ | -0.160^*^ |
|  | (0.066) | (0.071) | (0.067) |
| Slope | 0.199^***^ | 0.199^***^ | 0.196^***^ |
|  | (0.051) | (0.051) | (0.052) |
| Roads | -0.121^**^ | -0.115^**^ | -0.121^**^ |
|  | (0.040) | (0.041) | (0.040) |
| Agriculture |  | -0.036 |  |
|  |  | (0.052) |  |
| Aquiculture |  |  | -0.021 |
|  |  |  | (0.043) |
| Log Likelihood | 146.269 | 146.522 | 146.399 |
| AICc | -277.690 | -275.944 | -275.699 |
| Delta | 0.000 | 1.746 | 1.990 |
| Weight | 0.559 | 0.234 | 0.207 |
| Num. obs. | 140 | 140 | 140 |
| ^***^p < 0.001, ^**^p < 0.01, ^*^p < 0.05 | | | |

**Table 3**. Ranking of models for modeling scenario (c): prediction of ecological integrity at regional a scale, using human threat variables as predictors.

|  | **Model 1** | **Model 2** | **Model 3** | **Model 4** | **Model 5** | **Model 6** | **Model 7** |
| --- | --- | --- | --- | --- | --- | --- | --- |
| (Intercept) | 0.767^***^ | 0.762^***^ | 0.761^***^ | 0.782^***^ | 0.739^***^ | 0.762^***^ | 0.776^***^ |
|  | (0.024) | (0.024) | (0.024) | (0.027) | (0.020) | (0.024) | (0.026) |
| Roads | -1.576^***^ | -1.756^***^ | -1.597^***^ | -1.353^**^ |  | -1.028 | -1.472^**^ |
|  | (0.413) | (0.420) | (0.406) | (0.455) |  | (0.667) | (0.430) |
| Thermoelectric |  | 0.566 |  |  |  |  |  |
|  |  | (0.369) |  |  |  |  |  |
| Hydroelectric |  |  | 4.426 |  |  |  |  |
|  |  |  | (3.217) |  |  |  |  |
| Water withdrawals |  |  |  | -6.125 |  |  |  |
|  |  |  |  | (5.382) |  |  |  |
| Human settlements |  |  |  |  | -0.649^**^ | -0.301 |  |
|  |  |  |  |  | (0.182) | (0.288) |  |
| Aquiculture |  |  |  |  |  |  | -1.193 |
|  |  |  |  |  |  |  | (1.328) |
| Log Likelihood | 30.708 | 31.961 | 31.728 | 31.414 | 30.038 | 31.305 | 31.152 |
| AICc | -54.457 | -54.256 | -53.789 | -53.160 | -53.115 | -52.943 | -52.636 |
| Delta | 0.000 | 0.201 | 0.668 | 1.296 | 1.342 | 1.513 | 1.820 |
| Weight | 0.221 | 0.200 | 0.158 | 0.116 | 0.113 | 0.104 | 0.089 |
| Num. obs. | 29 | 29 | 29 | 29 | 29 | 29 | 29 |
| ^***^p < 0.001, ^**^p < 0.01, ^*^p < 0.05 | | | | | | | |

**Table 4**. Ranking of models for modeling scenario (d): prediction of ecological integrity at a regional scale, using human threat and environmental variables as predictors.

|  | **Model 1** | **Model 2** | **Model 3** | **Model 4** | **Model 5** | **Model 6** | **Model 7** | **Model 8** |
| --- | --- | --- | --- | --- | --- | --- | --- | --- |
| (Intercept) | 0.767^***^ | 0.762^***^ | 0.761^***^ | 0.743^***^ | 0.782^***^ | 0.739^***^ | 0.762^***^ | 0.776^***^ |
|  | (0.024) | (0.024) | (0.024) | (0.031) | (0.027) | (0.020) | (0.024) | (0.026) |
| Roads | -1.576^***^ | -1.756^***^ | -1.597^***^ | -1.462^**^ | -1.353^**^ |  | -1.028 | -1.472^**^ |
|  | (0.413) | (0.420) | (0.406) | (0.420) | (0.455) |  | (0.667) | (0.430) |
| Thermoelectric |  | 0.566 |  |  |  |  |  |  |
|  |  | (0.369) |  |  |  |  |  |  |
| Hydroelectric |  |  | 4.426 |  |  |  |  |  |
|  |  |  | (3.217) |  |  |  |  |  |
| Slope |  |  |  | 0.004 |  |  |  |  |
|  |  |  |  | (0.003) |  |  |  |  |
| Water withdrawals |  |  |  |  | -6.125 |  |  |  |
|  |  |  |  |  | (5.382) |  |  |  |
| Human settlements |  |  |  |  |  | -0.649^**^ | -0.301 |  |
|  |  |  |  |  |  | (0.182) | (0.288) |  |
| Aquiculture |  |  |  |  |  |  |  | -1.193 |
|  |  |  |  |  |  |  |  | (1.328) |
| Log Likelihood | 30.708 | 31.961 | 31.728 | 31.490 | 31.414 | 30.038 | 31.305 | 31.152 |
| AICc | -54.457 | -54.256 | -53.789 | -53.313 | -53.160 | -53.115 | -52.943 | -52.636 |
| Delta | 0.000 | 0.201 | 0.668 | 1.143 | 1.296 | 1.342 | 1.513 | 1.820 |
| Weight | 0.196 | 0.178 | 0.141 | 0.111 | 0.103 | 0.100 | 0.092 | 0.079 |
| Num. obs. | 29 | 29 | 29 | 29 | 29 | 29 | 29 | 29 |
| ^***^p < 0.001, ^**^p < 0.01, ^*^p < 0.05 | | | | | | | | |
|  | | | | | | | | |

|  |
| --- |

|  |
| --- |

**Table 5.** Best models for predicting the **weighted ecological integrity index** (EII) in four different modeling scenarios (a-d). All predictor variables, included environmental factors, were scaled between 0-1.

| Modeling scenarios | Selected variables | Coefficients | AICc | Mean R^2^ (from cross-validation) |
| --- | --- | --- | --- | --- |
| a. River EII prediction, with threat variables (N = 140) | *Intercept*  Human settlements  Roads | 0.684 ***  -0.135 *  -0.097 * | -251.8 | 0.1 (SD 0.11) |
| b. River EII prediction, with threat and environmental variables (N = 140) | *Intercept*  Human settlements  Roads  Oil activities  Elevation  Slope | 0.7 ***  -0.111  -0.119 **  -0.126  -0.177 ***  0.241*** | -271.5 | 0.21 (SD 0.13) |
| c. EII prediction with threat variables (N = 29) | *Intercept*  Human settlements | 0.673 ***  -0.583 * | -38.9 | 0.5 (SD 0.31) |
| d. Microbasin EII prediction with threat and environmental variables (N = 29) | *Intercept*  Human settlements | 0.673 ***  -0.583 * | -38.9 | 0.5 (SD 0.31) |

*** *p* < 0.001, ** *p* < 0.01, * *p* < 0.05
